# Oral Administration of *Lactobacillus rhamnosus* GG Ameliorates *Salmonella* Infantis-Induced Inflammation in a Pig Model via Activation of the IL-22BP/IL-22/STAT3 Pathway

**DOI:** 10.3389/fcimb.2017.00323

**Published:** 2017-07-18

**Authors:** Gui-Yan Yang, Jiao Yu, Jin-Hui Su, Lian-Guo Jiao, Xiao Liu, Yao-Hong Zhu

**Affiliations:** College of Veterinary Medicine, China Agricultural University Beijing, China

**Keywords:** *Salmonella* infantis, *Lactobacillus rhamnosus* GG, T-bet, IL-22, IL-22BP, CCL20, pig

## Abstract

The high rate of *Salmonella enterica* serovar Infantis (*S*. Infantis) infection poses significant risk for the development of non-typhoidal *Salmonella* gastroenteritis. However, efficient strategies to prevent or treat the infection remain elusive. Here, we explored the effect of the probiotic *Lactobacillus rhamnosus* GG (LGG) administration in preventing *S*. Infantis infection in a pig model. Probiotic LGG (1.0 × 10^10^ CFU/day) was orally administered to newly weaned piglets for 1 week before *S*. Infantis challenge. LGG pretreatment reduced the severity of diarrhea and alleviated intestinal inflammation caused by *S*. Infantis. Pre-administration of LGG excluded *Salmonella* from colonization of the jejunal mucosa but increased the abundance of *Bifidobacterium* in the feces. LGG promoted the expansion of CD4^+^ T-bet^+^ IFNγ^+^ T cells but attenuated *S*. Infantis-induced increases in the percentage of CD4^+^ IFNγ^+^ T cells and serum interleukin (IL)-22 levels in peripheral blood after *S*. Infantis challenge. In the small intestine, LGG pretreatment upregulated expression of the transcription factor T-bet but downregulated the *S*. Infantis-induced increase of CD4^+^ IFNγ^+^ T cells in Peyer's patches and IL-7Rα expression in the jejunum. Notably, LGG-treated pigs had enhanced expression of IL-22 and activated STAT3 in the ileum in response to *S*. Infantis infection. Pretreatment of pigs with LGG also elevated intestinal IL-22-binding protein production in response to *S*. Infantis challenge. In contrast, LGG consumption reduced the *S*. Infantis-induced increase in the number of CCL20-expressing cells in the jejunum. Our results suggest that the mechanism by which LGG ameliorates the intestinal inflammation caused by *S*. Infantis involves the upregulation of T-bet, activation of STAT3, and downregulation of CCL20.

## Introduction

Salmonellosis is a common disease in humans and animals worldwide, with high morbidity and mortality. About 93.8 million cases of human non-typhoidal *Salmonella* gastroenteritis occur annually, resulting in 155,000 deaths (Majowicz et al., 2010). *Salmonella enterica* serovar Infantis (*S*. Infantis), together with non-typhoidal *Salmonella* serovars Mbandaka, Bredeney, and Virchow, are more frequently associated with persistent salmonellosis than other serovars (Marzel et al., 2016). It was reported that *S*. Infantis was responsible for about 5% of human salmonellosis cases in Hungary in 2005–2006 (Nogrady et al., 2008). Infection with *S*. Infantis is characterized by diarrhea and fever, especially in infants aged ≤1 year (Bassal et al., 2014). *Salmonella*-infected pigs and pork products are important causes of human foodborne salmonellosis worldwide (Boyen et al., 2008). In recent years, *S*. Infantis has been one of the most prevalent serotypes in pigs and their environment (Keelara et al., 2013). As current treatments for non-typhoidal *S. enterica* infections using antimicrobial agents remain controversial, an efficacious strategy for prevention of salmonellosis would represent an important advance.

Using probiotics to harness the ability of the microbiota to affect host immunity is an important strategy for preventing intestinal bacterial infections (Ivanov and Honda, 2012). *Lactobacillus rhamnosus* GG (LGG) is the best studied member of the lactic acid bacteria and is known to have positive effects on human health (Floch, 2014). LGG was shown to be effective in the treatment of acute infectious diarrhea in children and in prevention of diarrhea caused by enterotoxigenic *Escherichia coli* in piglets (Szajewska et al., 2007; Zhang et al., 2010). Administration of a probiotic mixture of *Lactobacillus* spp. can alleviate disease signs of *S*. Typhimurium infection in weaned pigs (Casey et al., 2007), but several clinical trials have reported risks associated with LGG administration (Trevisi et al., 2011; Doron and Snydman, 2015). However, the exact mechanism underlying the activity of LGG against pathogenic microorganisms and its regulation of immune responses during *Salmonella* infection is poorly understood.

Oral administration of the *Lactobacillus plantarum* strain YU showed a beneficial effect in preventing viral infections by activating Th1 immune responses (Kawashima et al., 2011). LGG stimulation promotes the production of IFNγ in human mononuclear cells *in vitro* (Vaarala, 2003). T-bet^−/−^CD4^+^ T cells produce essentially normal amounts of IFNγ that controls the activation of *Salmonella*-infected macrophages for intracellular bacterial killing (Helmstetter et al., 2015). However, little is known regarding how LGG regulates T-bet^+/−^IFNγ^+/−^CD4^+^ T cells after *Salmonella* infection. In humans, elevated expression of T-bet in T cells from the lamina propria has been confirmed in patients with Crohn's disease and celiac disease (Matsuoka et al., 2004; Monteleone et al., 2004). In contrast, upregulation of T-bet mRNA expression by *Bifidobactrium longum* BB536 indirectly ameliorates colonic inflammation in ulcerative colitis (Takeda et al., 2009). *Bifidobacterium* BB12 and *L. plantarum* NCU116 upregulate the expression of T-bet protein in the small intestine of immunosuppressed mice (Xie et al., 2015).

Interleukin (IL)-22 plays a central role in host protection against pathogens at mucosal surfaces through the activation of signal transducer and activator of transcription 3 (STAT3) (Backert et al., 2014). IL-22-binding protein (IL-22BP), as a soluble binding receptor and antagonist of IL-22, can compete with membrane-bound IL-22 receptor 1 for the binding of free cytokine molecules and thus prevent IL-22 from generating a signal (Dumoutier et al., 2001). CD4^+^ T cell-derived IL-22BP demonstrated a pathogenic role in inflammatory bowel disease (IBD) (Pelczar et al., 2016). Orally fed *Bacillus* mixture was shown to upregulate IL-22 mRNA expression in the small intestine in response to *E. coli* challenge (Yang et al., 2016). Considering that IL-22 exhibits both protective and pathologic effects (Dudakov et al., 2015), the regulatory role of IL-22BP during *Salmonella* infection is uncertain.

IL-22-producing cells expressed with CC-chemokine receptor 6 (CCR6) are attracted by CC-chemokine ligand 20 (CCL20) into the tissues. In humans, the CCL20-CCR6 axis is involved in active inflammation in IBD, with enhanced expression of CCL20 in intestinal epithelial cells (Kaser et al., 2004; Skovdahl et al., 2015). Oral inoculation with *S. enteritidis* induces CCL20 production in the spleen of mice (Fahy et al., 2004). LGG was shown to significantly suppress *E. coli*-induced CCL20 expression in Caco-2 cells (Toki et al., 2009).

In the present study, we investigated the pathways through which probiotic LGG prevents salmonellosis, using a pig model of enteritis caused by *S*. Infantis. We hypothesized that oral administration of LGG would attenuate the intestinal inflammation via activation of the IL-22BP/IL-22/STAT3 pathway.

## Materials and methods

### Ethics statement

Animal care procedures were in strict accordance with the *Guidelines for Laboratory Animal Use and Care* from the Chinese Center for Disease Control and Prevention and the *Rules for Medical Laboratory Animals* from the Chinese Ministry of Health, under protocol CAU20151001-1, approved by the Animal Ethics Committee of China Agricultural University. Pigs were sacrificed under xylazine hydrochloride anesthesia, and every effort was made to minimize animal suffering.

### Animals

Twenty-one healthy (Landrace × Large White) piglets of mixed gender, selected from 8 different litters, weaned at 21 days of age, and weighing 6.29 ± 0.08 kg were obtained from Beijing Hog Raising and Breeding Center and used in this study. Each animal was penned separately and had access to an antibiotic-free feed and water *ad libitum* from day 0 (when newly weaned and transported to the animal experimental facility of the College of Veterinary Medicine, China Agricultural University) until day 18 (when euthanized).

Rectal temperature and other clinical signs were recorded daily. Severity of diarrhea was scored according to previously published criteria, with minimal modifications (Li et al., 2012). Piglets were considered to have mild diarrhea with a score of 4 and severe diarrhea when the score was 5 or 6.

### Characterization of the *S*. Infantis challenge strain

*Salmonella enterica* serovar Infantis strain (named as *S*. Infantis CAU1508) was isolated from the intestinal contents of weaned pigs with diarrhea. Briefly, the fresh sample was added to buffered peptone water (Beijing Aoboxing Bio-tech Co., Beijing, China) and incubated at 37°C for 16–18 h with shaking. A 0.3-ml aliquot of the pre-enrichment broth was transferred onto modified semi-solid Rappaport Vassiliads (MSRV; Beijing Land Bridge Technology Co., Beijing, China) agar and incubated for 24 h at 42 ± 1°C. After selective enrichment, an inoculating loop of white or gray colonies grown on MSRV was plated on xylose lysine tergitol 4 (XLT4; Beijing Land Bridge Technology Co., Beijing, China) agar and incubated at 37 ± 1°C overnight. Bacterial colonies showing morphologic characteristics of *Salmonella* were then confirmed by amplification of the *stn* gene by PCR and using the API 20E biochemical identification system (BioMérieux, Beijing, China). Serovar was determined by slide agglutination with commercial *Salmonella* antisera (Statens Serum Institute, Denmark) following the Kauffmann-White scheme. After identification and purification, a single colony of *S*. Infantis from an XLT4 agar plate was transferred and grown in Luria-Bertani (LB) broth (Oxoid, Basingstoke, England) overnight at 37°C with shaking and then stored at −70°C.

Next, *S*. Infantis CAU1508 harboring the plasmid pFPV-mCherry/2 (obtained from Olivia Steele-Mortimer [Addgene plasmid #20956]), which constitutively expresses mCherry under control of the *Salmonella rpsM* promoter, was prepared as previously described (Drecktrah et al., 2008).

We detected virulence factors associated with diarrhea or fever in the inoculated *S*. Infantis strain CAU1508 via real-time PCR assay as previously described (Zhou et al., 2015). The virulence factors *SipA, SipB, SopA, SopB, SopD, SopE, SopE2, FljB*, and *SseI* were found in this strain, whereas *FljA* was absent. The primers used are listed in Table S1.

### Bacteria preparation and growth conditions

*Lactobacillus rhamnosus* GG (ATCC 53103) freeze-dried powder (Gefilus, Valio Ltd., Helsinki, Finland) was used to prepare LGG solution, as previously described (Zhang et al., 2010). Briefly, LGG was grown in De Man, Rogosa, and Sharpe (MRS) broth (Oxoid, Hampshire, UK) overnight at 37°C under microaerophilic conditions. Bacteria were pelleted by centrifugation at 3,000 × *g* for 10 min at 4°C and resuspended in physiological saline. The concentration of LGG was adjusted to 10^9^ CFU/ml.

The *S*. Infantis CAU1508 (mCherry) challenge strain was grown to mid-log phase in fresh LB broth at 37°C with shaking. The *Salmonella* were then harvested by centrifugation at 3,000 × *g* for 10 min at 4°C, washed 3 times with sterile physiologic saline, and resuspended in physiologic saline. An inoculum of *S*. Infantis CAU1508 (mCherry) containing 5.0 × 10^10^ CFU/ml was prepared and quantified by determination of CFUs after plating the serial dilutions of bacterial suspensions onto XLT4 agar plates.

### Experimental design

On the day of weaning (day 0), pigs were divided into 3 groups (*n* = 7 per group) according to weight and ancestry: (1) control (CN) group (oral administration of sterile physiological saline only); (2) *S*. Infantis (SI) group (oral administration of sterile physiological saline and *S*. Infantis); and (3) LGG + *S*. Infantis (LS) group (oral administration of LGG and *S*. Infantis). All animals were administered the test material orally, as previously described (Yang et al., 2016), and were determined to be free of *Salmonella* by analysis of feces before the study.

At 9:00 a.m. from days 1 to 7, pigs in group LS were administered 10 ml of LGG (10^9^ CFU/ml) intragastrically, whereas pigs in groups CN and SI were administered 10 ml of sterile physiological saline daily. At 9:00 a.m. on day 8, pigs in groups SI and LS were orally inoculated with 10 ml of *S*. Infantis (5.0 × 10^10^ CFU/ml), whereas CN pigs received 10 ml of sterile physiological saline.

To assess bacterial colonization, fresh fecal samples were collected on days 1, 4, 8, 13, and 16 after weaning. Blood samples were collected from the jugular vein of pigs prior to *S*. Infantis challenge (0 h) and at 6, 12, 24, 48, 96, 144, and 192 h post-challenge. A 3-ml aliquot of peripheral blood collected in Venoject glass tubes containing EDTA (Terumo Europe NV, Leuven, Belgium) at 0, 24, and 192 h following *S*. Infantis challenge was used for flow cytometry analysis. Blood samples without additives were centrifuged, and the serum was stored at −80°C for the determination of peripheral levels of IL-7 and IL-22.

On day 10 post-infection, pigs were euthanized and tissue samples were immediately collected. The middle jejunum (without Peyer's patch [PP] involvement) and ileum segments collected from each pig were flash frozen in liquid nitrogen and then stored at −80°C for later mRNA and Western blot analyses. For immunostaining studies, the proximal, mid-, and distal segments of the jejunum and ileum (approximately 10 × 15 × 3 mm) were fixed in 4% paraformaldehyde and embedded in paraffin. For immunofluorescence studies, the specimens were embedded in OCT (optimal cutting temperature) compound during the freezing process.

### Bacterial enumeration

One gram of feces or mucosal tissues from each animal was homogenized in 9 ml of sterile saline solution, and serial dilutions were then plated on XLT4 agar plates for *Salmonella* culture and MRS agar plates for lactobacilli culture (Beijing Land Bridge Technology Co., Beijing, China). XLT4 agar plates were incubated for 24 h at 37°C under aerobic conditions, whereas MRS agar plates were incubated under anaerobic conditions for 48 h at 37°C. Results are expressed as log_10_ CFU/g feces or log_10_ CFU/g mucosal tissues. All counts were performed in triplicate.

### PCR quantification of 16S rRNA genes

Quantitative PCR was performed as previously described, with minor modifications (Yang et al., 2016). The following primer sets were used: “all bacteria,” 5′-CGGTGAATACGTTCCCGG-3′, 5′-TACGGCTACCTTGTTACGACTT-3′; *Escherichia coli*: 5′-CATGCCGCGTGTATGAAGAA-3′, 5′-CGGGTAACGTCAATGAGCAAA-3′; *Bifidobacterium*: 5′-CGGGTGAGTAATGCGTGACC-3′, 5′-TGATAGGACGCGACCCCA-3′. The results for all samples were normalized to the 16S rRNA gene level of “all bacteria.”

### Enzyme-linked immunosorbent assay (elisa)

The serum concentrations of IL-7 and IL-22 were determined by direct high-sensitivity sandwich ELISA specific for porcine IL-7 (LifeSpan Biosciences, Seattle, WA) or porcine IL-22 (LifeSpan Biosciences), respectively. The concentrations of TNF-α and IL-1β in mucosal tissues of the jejunum and ileum were measured by porcine-specific commercially available ELISA kits (Westang Biotechnology, Shanghai, China). The experimental procedure was based on the manufacturer's instructions, with minimal modifications.

### Histologic assessment

Intestinal pathology was evaluated on hematoxylin & eosin-stained jejunal and ileal sections by a single blinded scorer using a validated scoring system (Zhou et al., 2015). Histologic grading was based on observed epithelial integrity, central lacteal expansion, leukocyte infiltration, submucosal edema, mucosal hyperemia, and lymphoid necrosis of PPs. Each segment was given a score ranging from 0 to 3. For epithelial integrity: grade 0, no change; 1, shedding of <10 epithelial cells per lesion; 2, shedding of 11–20 epithelial cells per lesion; and 3, epithelial ulceration. For central lacteal expansion: 0, no change; 1, mild; 2, moderate; and 3, profound. For leukocyte infiltration: 0, <10 leukocytes per field; 1, 11–15 leukocytes per field; 2, 16–20 leukocytes per field; and 3, >20 leukocytes per field. For submucosal edema: 0, no change; 1, mild; 2, moderate; and 3, profound. For mucosal hyperemia: 0, none; 1, mild; 2, moderate; and 3, severe. For lymphoid necrosis of PPs: 0, none; 1, mild; 2, moderate; and 3, severe. Scores for each criterion were added to give an overall inflammation score of 0–15 for jejunal samples or 0–18 for ileal samples.

### Immunofluorescence

Attachment of *S*. Infantis to the intestinal mucosa was determined by an indirect immunofluorescence assay. Tissue sections (6 μm) obtained from the jejunum and ileum were incubated with monoclonal rabbit anti-pig α-tubulin (1:200 dilution, ab52866; Abcam, Cambridge, UK) overnight at 4°C. The secondary antibody was goat anti-rabbit fluorescein isothiocyanate [FITC]-conjugated (ZF-0311; Zhongshan Golden Bridge Biotechnology, Beijing, China), and DAPI (4′,6′-diamidino-2-phenylindole; Sigma-Aldrich) was used for nuclear staining. The slides were visualized and photographed using a Nikon Eclipse Ti-U inverted fluorescence microscope equipped with a Nikon DS cooled camera head (Nikon).

### Flow cytometry

Single-cell suspensions from peripheral blood and the intestine were isolated as previously described (Zhu et al., 2014). Cells were stimulated *in vitro* by incubation with 50 ng/ml of phorbol myristate acetate, 500 ng/ml ionomycin, and BD GolgiPlug (BD PharMingen) in complete RPMI 1640 medium at 37°C for 4 h. The monoclonal antibodies used were as follows: mouse anti-pig CD3ε (clone P2G10, FITC-conjugated, 559582; BD Biosciences), mouse anti-pig CD4α (clone 74-12-4, PerCP-Cy5.5-conjugated, 561474; BD Biosciences), mouse anti-human T-bet (clone O4-46, phycoerythrin [PE]-conjugated, 561268; BD Biosciences), and mouse anti-pig IFN-γ (clone P2G10, AlexaFluor 647-conjugated, 561480; BD Biosciences). Isotype controls PerCP-Cy5.5-conjugated mouse IgG2b, κ (558304; BD Biosciences), PE-conjugated mouse IgG1, κ (559320; BD Biosciences) and AlexaFluor 647-conjugated mouse IgG1, κ (557732; BD Biosciences) were included. The stained cells were analyzed on a FACScalibur™ flow cytometer (BD Biosciences), and data analysis was performed using FlowJo 9.3 software (Tree Star).

### Quantitative real-time PCR

Total RNA was extracted from frozen tissues using an EASYspin plus RNA extraction kit (Aidlab Biotechnologies, Beijing, China) according to the manufacturer's instructions. Reverse transcription and real-time PCR were performed as previously described (Yang et al., 2016). Data were normalized to the geometric mean *C*_*T*_ values of three reference genes: β-actin, glyceraldehyde-3-phosphate dehydrogenase (GAPDH) and hypoxanthine phosphoribosyl-transferase. Results are presented as fold change using the 2^−ΔΔCT^ method, as previously described (Zhou et al., 2015). Primers are listed in Table S2.

### Western blotting

Proteins were extracted from the jejunum and ileum using Radio-Immunoprecipitation Assay buffer (RIPA; Sigma-Aldrich, St. Louis, MO), as previously described (Yang et al., 2016). The primary antibodies used were polyclonal rabbit anti-pig IL-22 (1:1,500 dilution, ab193813; Abcam), polyclonal rabbit anti-human STAT3 (1:1,000 dilution, AP0365; Bioworld Technology, Nanjing, China), polyclonal rabbit anti-human p-STAT3 (phospho-S727) (1:1,000, AP0248; Bioworld Technology), monoclonal mouse anti-human IL-22RA2 (1:1,500 dilution, 66190-1-Ig; Proteintech, Chicago, IL), polyclonal rabbit anti-human IL-7Rα (1:5,000 dilution, ab115249; Abcam), and monoclonal mouse anti-GAPDH (1:2,000 dilution, 60004-1-Ig; Proteintech). Horseradish peroxidase-conjugated secondary antibodies used were goat anti-rabbit IgG (H+L) (SA00001-2; Proteintech), goat anti-mouse IgG (H+L) (SA00001-1;Proteintech), and rabbit anti-goat IgG (H+L) (ZB-2306; Golden Bridge Biological Technology). Immobilon Western chemiluminescent HRP substrate (Millipore, Billerica, MA) was used for immunoblot detection. The bands were visualized using a Tanon-5200 gel image system (Tanon, Shanghai, China). The intensity of bands was quantified by densitometric analysis using ImageJ software (National Institutes of Health, Bethesda, MD). Results are presented as the ratio of the intensity of the IL-22, IL-22BP, or IL-7Rα band to that of the GAPDH band or the ratio of the intensity of the p-STAT3 band to that of the STAT3 band.

### Immunohistochemistry

The jejunal and ileal tissue samples embedded in paraffin were sectioned at 3-μm thickness, and immunohistochemistry was performed using the un-avidin-biotin complex technique, a highly sensitive system (Envision polymer-system, Biogenex, San Ramon, CA). Briefly, tissue sections were boiled in 10 mM citrate buffer (pH 6.0) for antigen retrieval. Slides were quenched with 3% hydrogen peroxide solution for 10 min at room temperature. After a washing procedure with phosphate-buffered saline (PBS), the slides were incubated with goat anti-pig CCL20 polyclonal primary antibody (1:400 dilution, MBS421719; MyBioSource, San Diego, CA) or mouse anti-human IL-22BP monoclonal primary antibody (1:1,000 dilution, 66190-1-Ig; Proteintech) in a humidified chamber overnight at 4°C. After 3 rinses in PBS, the slides were incubated with the secondary horseradish peroxidase-conjugated anti-goat IgG or anti-mouse IgG (unbiotinylated antibodies, Envision System, DakoCytomation) for 20 min at 37°C. Tissue staining was visualized with 3,3′-diaminobenzidine (Zhongshan Golden Bridge Biotechnology). Negative controls in which PBS and goat serum or mouse serum replaced the primary antibody during incubation were included in each batch. Images were captured using an Olympus BX41 microscope (Olympus, Tokyo, Japan) equipped with a Canon EOS 550D camera head (Canon, Tokyo, Japan). The detection of CCL20 was executed through a ranked score of 0–4, which was used to evaluate the number of positive cells per section taken from each block, as previously described (Li et al., 2014). The indications for the scores were as follows: 0 = no positive cells, 1 = 1–10 positive cells, 2 = 11–30 positive cells, 3 = 31–50 positive cells, and 4 = or > 50 positive cells. The number of CCL20-positive cells in the jejunum of each pig was scored from 10 fields at a magnification of ×400.

### Statistical analysis

The SAS statistical software package, version 9.3 (SAS Institute Inc., Cary, NC) and the software's PROC MIXED procedure were used for statistical analyses. The incidence of diarrhea was analyzed using Pearson's chi-squared test. For non-normally distributed and repeated-measure data analysis, the non-parametric Friedman's test using the SAS procedure FREQ was performed to compare diarrhea scores between treatments. Moreover, the non-parametric Wilcoxon-Mann-Whitney *U*-test was performed to compare differences between treatments in histologic scores for the small intestine. Differences between least-square means were compared using Tukey's test. Data are presented as the mean ± standard error of the mean (SEM). *P*-values: ^*^P < 0.05; ^**^P < 0.01; ^***^P < 0.001.

## Results

### Clinical signs

**Figure 1:**
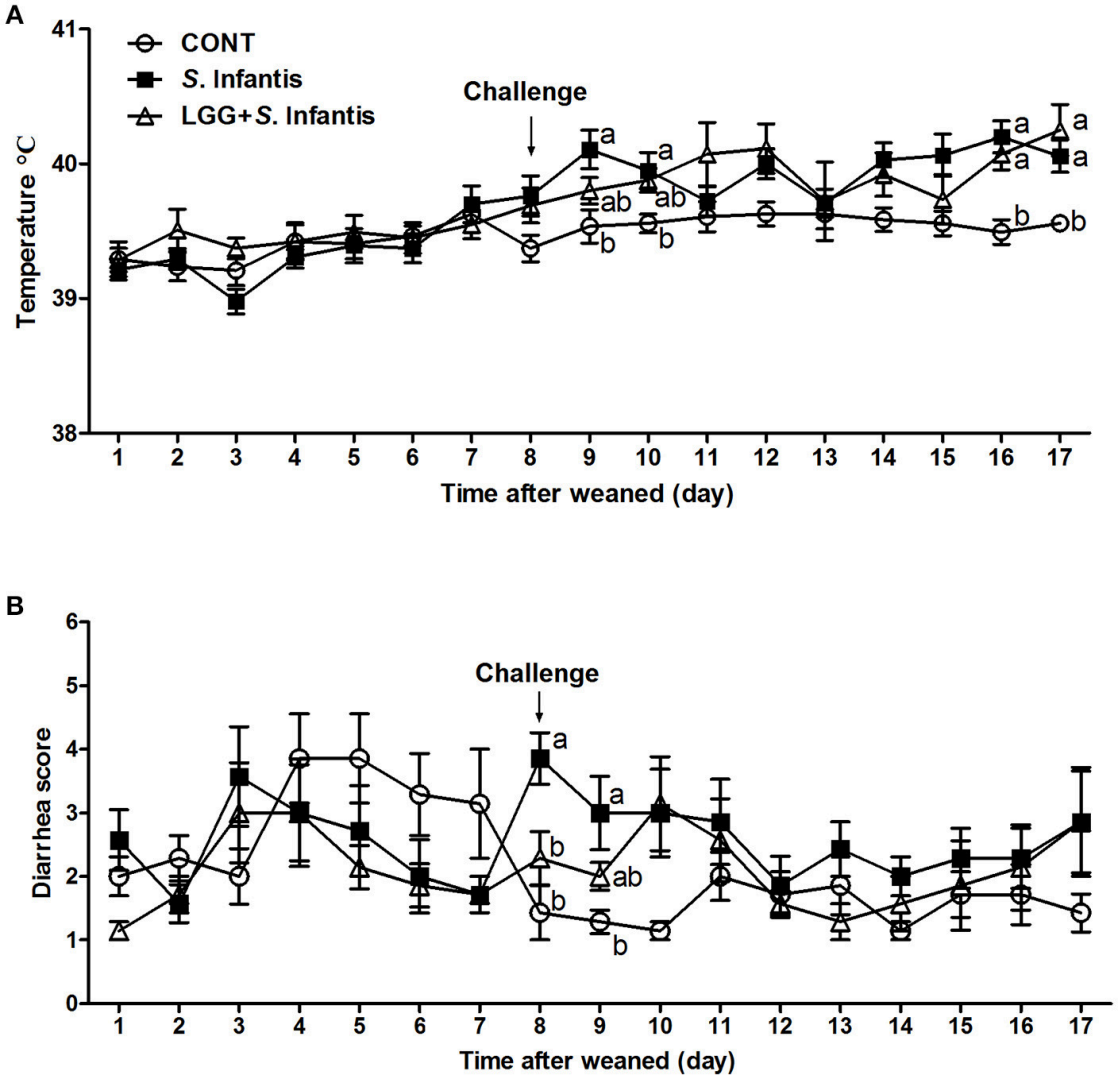
Effect of LGG on clinical signs of *S*. Infantis infection. **(A)** Rectal temperature and **(B)** diarrhea scores in pigs (*n* = 7 per group) that received sterile physiological saline orally (CONT), sterile physiological saline orally followed by *S*. Infantis (5.0 × 10^10^ CFU/ml, 10 ml, p.o.) challenge (*S*. Infantis), or were pretreated with LGG (1.0 × 10^9^ CFU/ml, 10 ml once daily, p.o.) for 1 week followed by *S*. Infantis challenge (LGG + *S*. Infantis). Mean values at the same time point without a common superscript (^a, b^) differ significantly (*P* < 0.05; Tukey's test).

The rectal temperature in all pigs was 39.38 ± 0.31°C before *S*. Infantis challenge. On day 9 (day 2 after *S*. Infantis challenge), the rectal temperature in *S*. Infantis-challenged pigs without LGG pretreatment increased to 40.11 ± 0.14°C compared with CONT pigs (*P* = 0.011; Figure 1A). On days 16 and 17, pigs pretreated with LGG also exhibited a higher temperature than CONT pigs.

The diarrhea scores were monitored for 17 days. During week 1 after weaning, 33.33% (7/21) of piglets experienced naturally acquired severe diarrhea (weaning diarrhea) lasting for 2 days due to stress. However, the mean diarrhea scores were lower in pigs pretreated with LGG than in CONT pigs (*P* = 0.001; Table S3). Within 24 h following *S*. Infantis challenge (day 8), 3 pigs in the *S*. Infantis treated group experienced severe watery diarrhea (fecal scores ≥5). In contrast, pigs pretreated with LGG had lower diarrhea scores compared with pigs infected only with *S*. Infantis (*P* = 0.040; Figure 1B). On day 9 post-challenge, *S*. Infantis treated pigs continued to exhibit more severe diarrhea than CONT pigs (*P* = 0.012; Figure 1B). During *S*. Infantis infection, the incidence of diarrhea in *S*. Infantis treated pigs was higher than that in CONT pigs (*P* < 0.001; Table S3); however, a similar difference compared with CONT pigs was also observed in pigs pretreated with LGG (*P* = 0.001).

### Orally fed LGG reduces colonization of the jejunum by *Salmonella*

**Figure 2:**
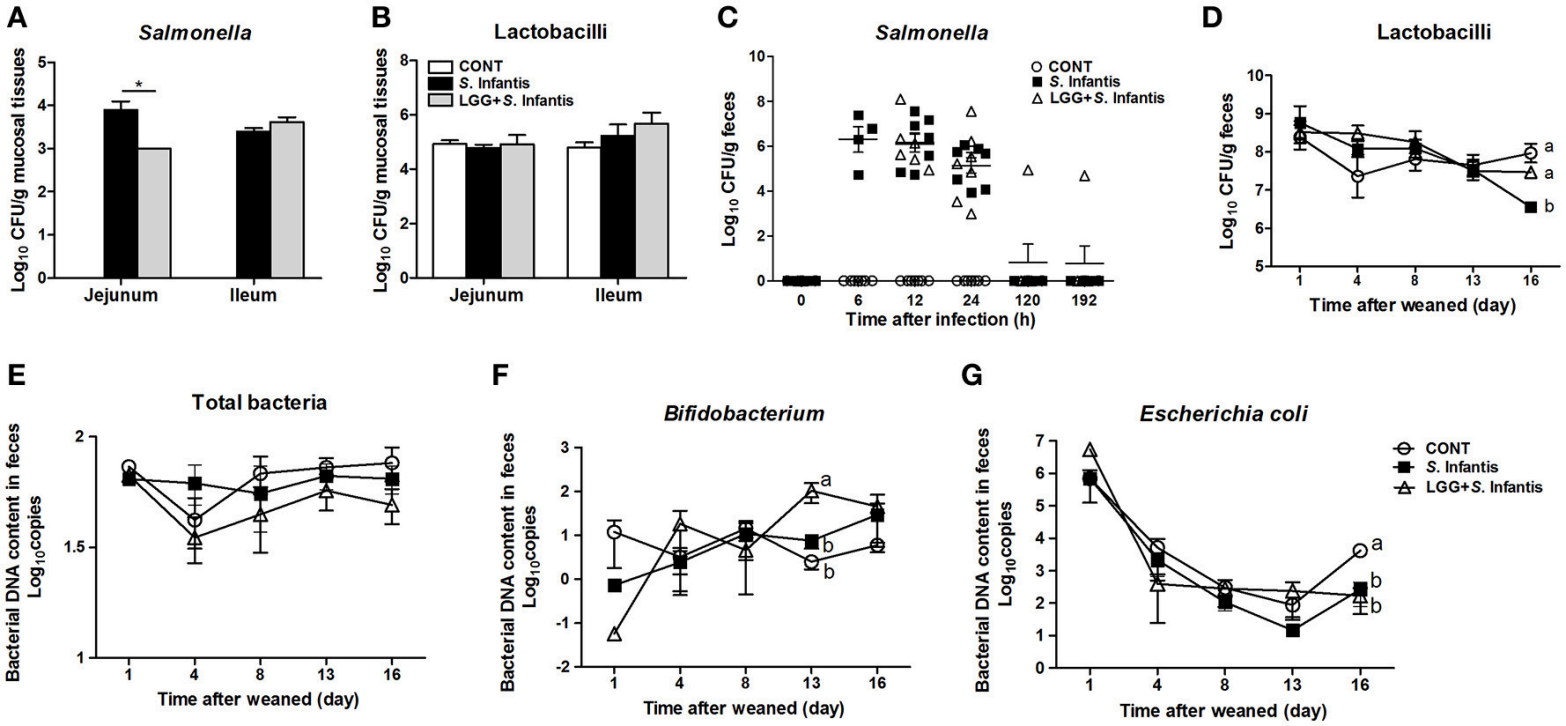
Orally fed LGG reduces the number of *Salmonella* in the jejunal mucosa but increases the fecal abundance of *Bifidobacterium* during *S*. Infantis infection. Fresh fecal samples from animals of the indicated groups (*n* = 6 per group) were collected on days 1, 4, 8 (0, 6, 12, and 24 h after infection), 13, and 16 after weaning. The mucosal tissues of the midjejunum and distal ileum were collected from the indicated pigs 10 days after *S*. Infantis challenge. The numbers of *Salmonella* **(A,C)** and Lactobacilli **(B,D)** in intestinal mucosal tissues or fecal shedding after *S*. Infantis challenge were monitored using culture-based enumeration. Bacterial DNA isolated from 200 mg of feces from pigs of the four groups was analyzed by quantitative PCR using universal primers for bacterial 16S rRNA genes **(E–G)**. Data are presented as means ± SEM. ^*^P < 0.05; Mean values at the same time point without a common superscript letter differ significantly (Tukey's test).

Pigs pretreated with LGG had fewer *Salmonella* in the mucosal tissues of the jejunum after *S*. Infantis infection, than did pigs only challenged with *S*. Infantis (*P* = 0.018, Figure 2A). However, no changes in the number of lactobacilli in jejunal and ileal mucosa were observed (Figure 2B). *S*. Infantis shedding occurred rapidly over a short time period (within approximately 2 days) (Figure 2C). By contrast, the number of lactobacilli in the feces of *S*. Infantis-challenged pigs was lower than in pigs pretreated with LGG and CONT pigs on day 16 (*P* = 0.028 and *P* < 0.001, respectively; Figure 2D). As expected, orally fed LGG increased the abundance of fecal *Bifidobacterium* compared with CONT and pigs infected only with *S*. Infantis on day 13 after infection (*P* = 0.018 and *P* = 0.034, respectively; Figure 2F). The amount of *E. coli* shed by pigs after *S*. Infantis infection was less than that of CONT pigs (*P* < 0.01; Figure 2G). No changes in the abundance of total bacteria in feces were detected in the present study (Figure 2E).

### Orally fed LGG ameliorates the intestinal inflammation caused by *S*. Infantis

**Figure 3:**
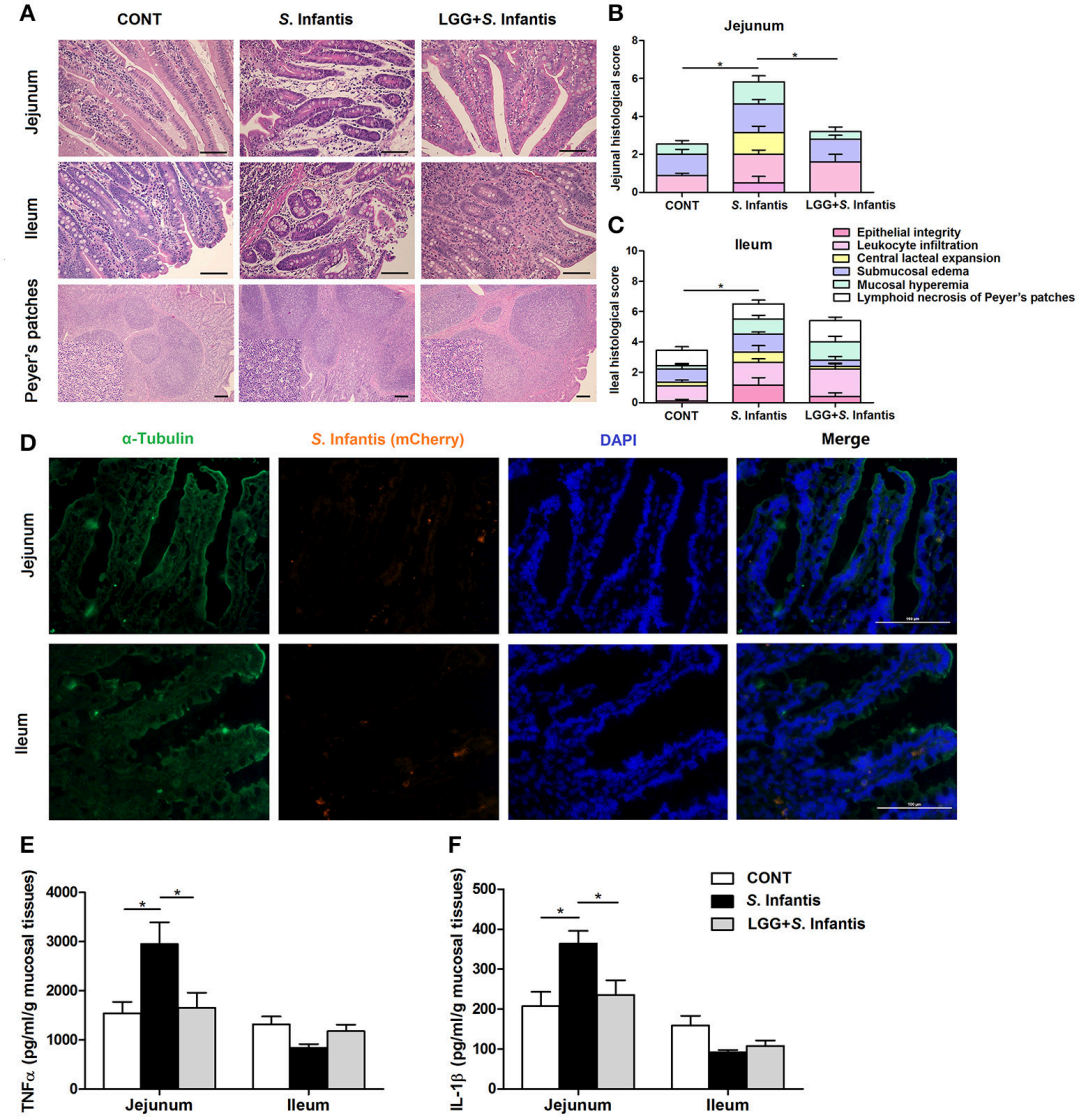
Orally fed LGG ameliorates intestinal inflammation in newly weaned pigs infected with *S*. Infantis. **(A)** Representative photomicrographs of hematoxylin and eosin-stained jejunal and ileal sections. Scale bars, 100 μm. **(B,C)** Jejunal and ileal histologic scores (non-parametric Wilcoxon-Mann-Whitney *U*-test). **(D)** Immunofluorescence analysis of the colonization of *S*. Infantis (mCherry) in the jejunum and ileum. Frozen sections of intestinal tissues from *S*. Infantis pigs were stained with anti-pig α-tubulin (green) and DAPI (blue). Scale bars, 100 μm. **(E,F)** The levels of TNF-α and IL-1β in mucosal tissues of the jejunum and ileum were detected by ELISA. Data are presented as the mean ± SEM for each tissue (*n* = 7 per group). ^*^P < 0.05 (Tukey's test).

On day 18 (10 days after *S*. Infantis challenge), histologic analyses showed that infection with *S*. Infantis caused epithelial lesions, central lacteal expansion, substantial leukocyte infiltration, submucosal edema, and mucosal hyperemia in the small intestine, as well as lymphoid necrosis of PPs (Figure 3A). Histologic assessment of the jejunum and ileum from pigs infected only with *S*. Infantis revealed more severe inflammation relative to that of CONT pigs (*P* = 0.011 and *P* = 0.037, respectively; Figures 3B,C), whereas LGG pretreatment tempered the severity of *Salmonella*-associated inflammation.

Colonization by *S*. Infantis (mCherry) was observed in the intestinal mucosa but not in the liver or spleen (Figure 3D).

The production of TNF-α and IL-1β in the jejunal mucosa of pigs was increased by challenge with *S*. Infantis compared with the CONT (*P* = 0.031 and *P* = 0.026, respectively; Figures 3E,F), but not in that of pigs pretreated with LGG.

### Orally fed LGG induces the expansion of CD4^+^ T-bet^+^ IFNγ^+^ T cells in peripheral blood

**Figure 4:**
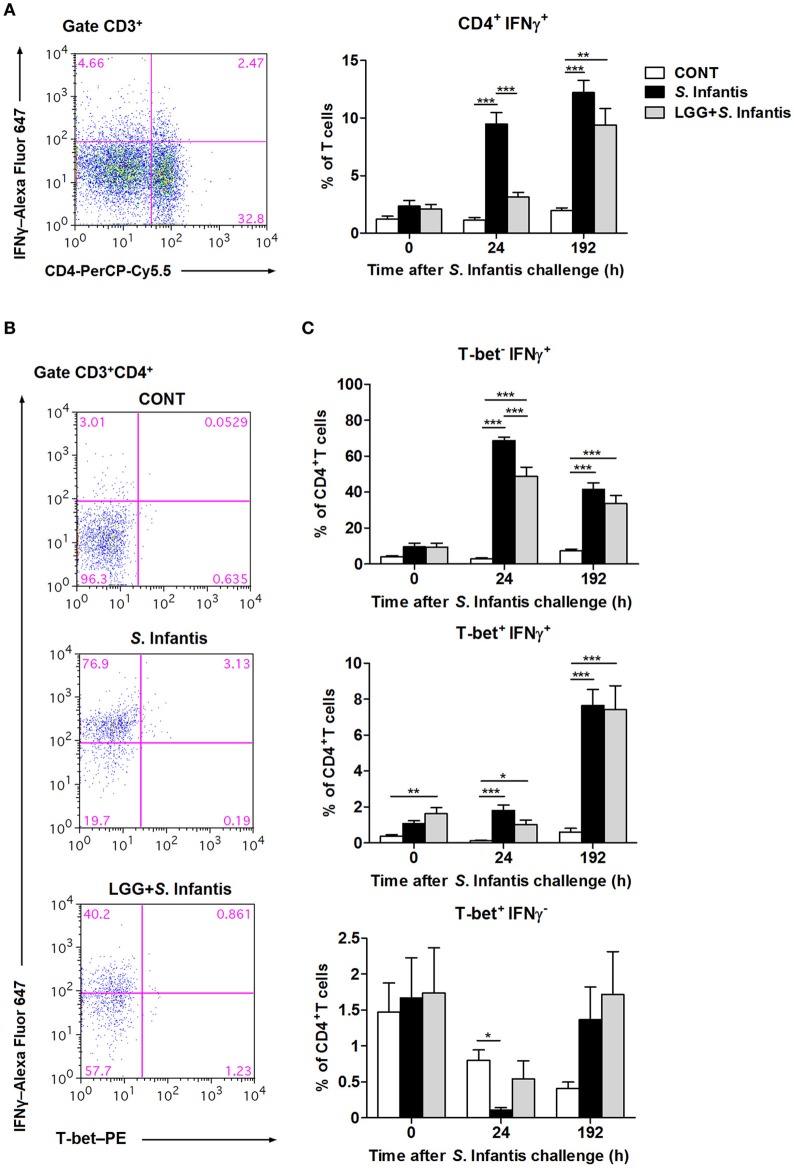
Orally fed LGG induces the expansion of CD4^+^ T-bet^+^ IFNγ^+^ T cells in peripheral blood. Peripheral blood samples were collected from the indicated pigs at 0, 24, and 192 h after *S*. Infantis challenge. **(A)** Flow cytometry analysis of the percentage of CD4^+^ IFNγ^+^ cells among CD3^+^ T cells. Left, representative flow cytometry dot plot shows the gating strategy for peripheral CD3^+^ T cells. **(B)** Representative dot plots show the percentages of T-bet^−/^^+^ IFNγ^−/^^+^ cells among CD4^+^ T cells at 24 h after infection. Flow cytometry analysis of the percentages of **(C)** T-bet^−^ IFNγ^+^, T-bet^+^ IFNγ^+^, and T-bet^+^ IFNγ^−^ cells within the peripheral CD4^+^ T-cell population in the indicated pigs. Data are presented as the mean ± SEM for each time point (*n* = 7 per group). ^*^P < 0.05; ^**^P < 0.01; ^***^P < 0.001 (Tukey's test).

To examine the effect of LGG on modulating systemic T cell-mediated immunity to *S*. Infantis, we examined the percentage of CD3^+^ CD4^+^ IFNγ^+^ T cells in peripheral blood at 0, 24, and 192 h following *S*. Infantis challenge. At 24 h after *S*. Infantis challenge, the percentage of peripheral blood CD4^+^ IFNγ^+^ T cells in pigs without LGG pretreatment was higher than that in CONT pigs (*P* < 0.001; Figure 4A). At 192 h post-challenge with *S*. Infantis, the percentage of peripheral blood CD4^+^ IFNγ^+^ T cells was higher than CONT, with or without LGG pretreatment (*P* < 0.01 and *P* < 0.001, respectively).

To further explore the role of the transcription factor T-bet in regulating IFNγ expression among CD4^+^ T cells, CD3^+^ CD4^+^ T-bet^+^^/−^ IFNγ^+^^/−^ T cells were also examined, as described above. Representative results at 24 h after *S*. Infantis challenge are shown in Figure 4B. Remarkably, at 24 h after challenge, the percentage of CD4^+^ T-bet^−^ IFNγ^+^ T cells in the peripheral blood of pigs pretreated with LGG was lower than in pigs without pretreatment (*P* < 0.001; Figure 4C). Orally fed LGG induced an increase in the proportion of CD4^+^ T-bet^+^ IFNγ^+^ T cells in peripheral blood before and after *S*. Infantis challenge compared with CONT pigs (*P* < 0.05; Figure 4C). Interestingly, the percentage of CD4^+^ T-bet^+^ IFNγ^−^ T cells decreased at 24 h after *S*. Infantis challenge in pigs not fed LGG compared with CONT pigs (*P* = 0.013; Figure 4C).

### Orally fed LGG suppresses the expression of IFNγ mRNA but elevates the expression of T-bet in the intestine during *S*. Infantis infection

**Figure 5:**
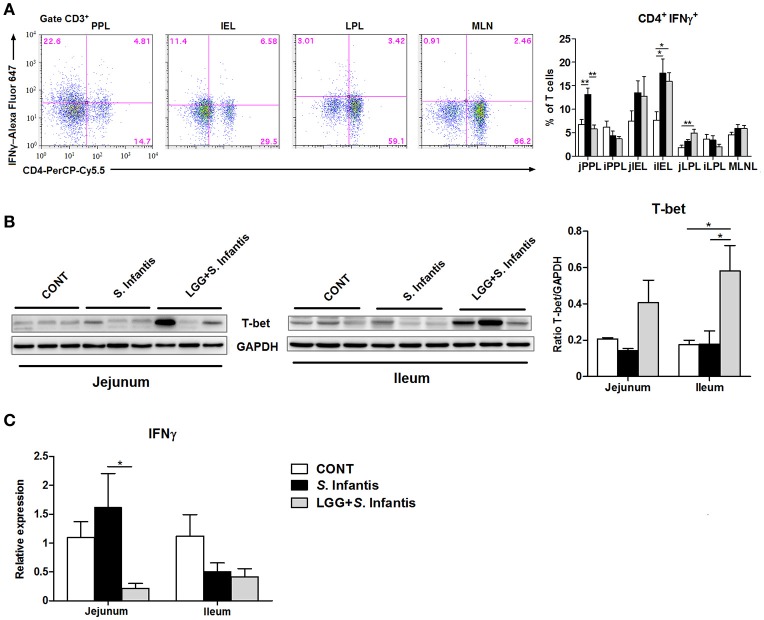
Orally fed LGG suppresses the expression of IFNγ mRNA but elevates the expression of T-bet in the intestine during *S*. Infantis infection. Peyer's patch lymphocytes (PPLs), intraepithelial lymphocytes (IELs), and lamina propria lymphocytes (LPLs) were collected from jejunal and ileal tissues from the indicated pigs 10 days after *S*. Infantis challenge. **(A)** Left, representative dot plots show the gating strategy for gut CD3^+^ T cells. Right, flow cytometry analysis of the percentage of CD4^+^ IFNγ^+^ cells among CD3^+^ T cells in the small intestine. **(B)** Representative Western blot results for T-bet in the small intestine collected from pigs 10 days after *S*. Infantis challenge (left panel). Each band represents a single pig. Results are presented as the ratio of the T-bet band intensity to the intensity of the GAPDH band (right panel). **(C)** Expression of IFNγ mRNA in both jejunal and ileal tissues collected from pigs 10 days after *S*. Infantis challenge was analyzed using quantitative real-time PCR. Data are presented as the mean ± SEM for each tissue (*n* = 6–7 per group). ^*^P < 0.05; ^**^P < 0.01 (Tukey's test).

We assessed changes in the proportion of CD4^+^ IFNγ^+^ T cells in the intestinal compartments, including the PPs, intraepithelial layer, and lamina propria of the jejunum and ileum. In the jejunum, the percentage of CD4^+^ IFNγ^+^ T cells from the PPs was significantly higher in pigs challenged with *S*. Infantis than in CONT pigs (*P* = 0.003), whereas this increase was attenuated by oral feeding of LGG (*P* = 0.001; Figure 5A). Similarly, the expression of IFNγ mRNA in the jejunum was downregulated in pigs pretreated with LGG compared with pigs challenged only with *S*. Infantis (*P* = 0.047; Figure 5C). Among jejunal lamina propria lymphocytes, there was a higher percentage of CD4^+^ IFNγ^+^ T cells in pigs pretreated with LGG than in CONT pigs (*P* = 0.007; Figure 5A). In the ileum, an expansion of CD4^+^ IFNγ^+^ T cells among intraepithelial lymphocytes was found in pigs infected with *S*. Infantis compared with CONT pigs (*P* < 0.05). Intriguingly, the expression of the transcription factor T-bet in the ileum was elevated in pigs pretreated with LGG compared with CONT pigs and pigs challenged only with *S*. Infantis (*P* = 0.048 and *P* = 0.05, respectively; Figure 5B).

### Orally fed LGG attenuates the *S*. Infantis-induced expression of IL-7Rα in the intestine

**Figure 6:**
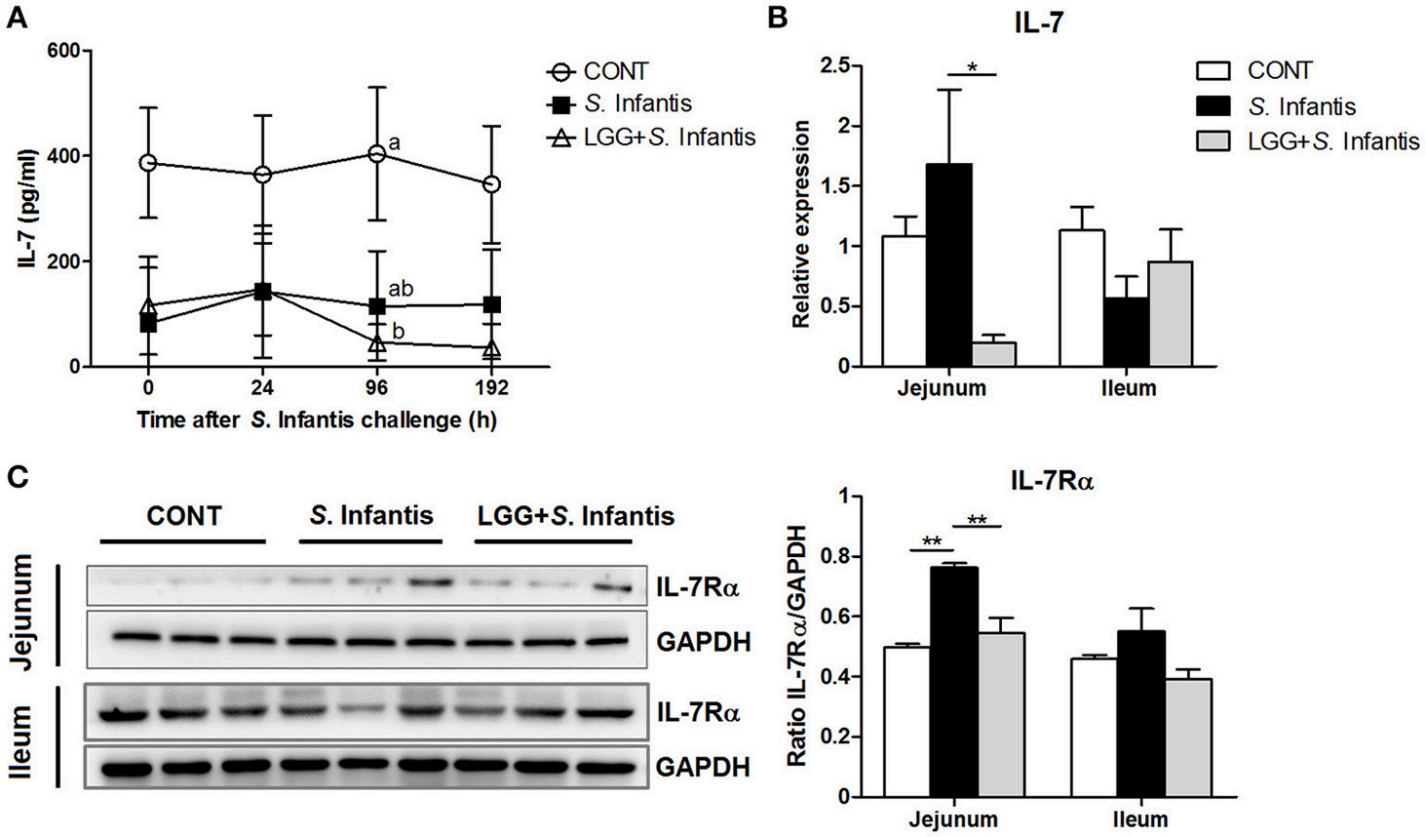
Effect of orally fed LGG on the expression of IL-7/IL-7Rα. **(A)** Serum concentrations of IL-7 were determined by ELISA. Data are presented as the mean ± SEM for each time point (*n* = 6 per group). Mean values at the same time point without a common superscript (^a, b^) differ significantly (*P* < 0.05). **(B)** Expression of IL-7 mRNA in both jejunal and ileal tissues collected from pigs 10 days after *S*. Infantis challenge was analyzed using quantitative real-time PCR. **(C)** Representative Western blot panels for IL-7Rα in jejunal and ileal tissues collected from pigs 10 days after *S*. Infantis challenge (left panel). Each band represents a single pig. Results are presented as the ratio of the IL-7Rα band intensity to the intensity of the GAPDH band (right panel). Data are expressed in arbitrary units as the mean ± SEM for each tissue (*n* = 6–7 per group). ^*^P < 0.05; ^**^P < 0.01 (Tukey's test).

IL-7 is essential for immune cell development and homeostasis. The levels of IL-7 in the serum of pigs pretreated with LGG were lower than those of CONT pigs at 96 h after *S*. Infantis challenge (*P* = 0.046; Figure 6A). The expression of IL-7 mRNA in the jejunum of pigs pretreated with LGG was lower than that in pigs only challenged with *S*. Infantis (*P* = 0.041; Figure 6B). The simultaneous expression of IL-7Rα (the receptor for IL-7) in the jejunum was higher in pigs challenged with *S*. Infantis compared with CONT pigs (*P* = 0.002), and this increase was attenuated by oral administration of LGG (*P* = 0.007; Figure 6C). No changes in the expression of IL-7 and IL-7Rα in the ileum were observed following *S*. Infantis challenge.

### Orally fed LGG activates STAT3 and elevates the expression of IL-22BP during *S*. Infantis infection

**Figure 7:**
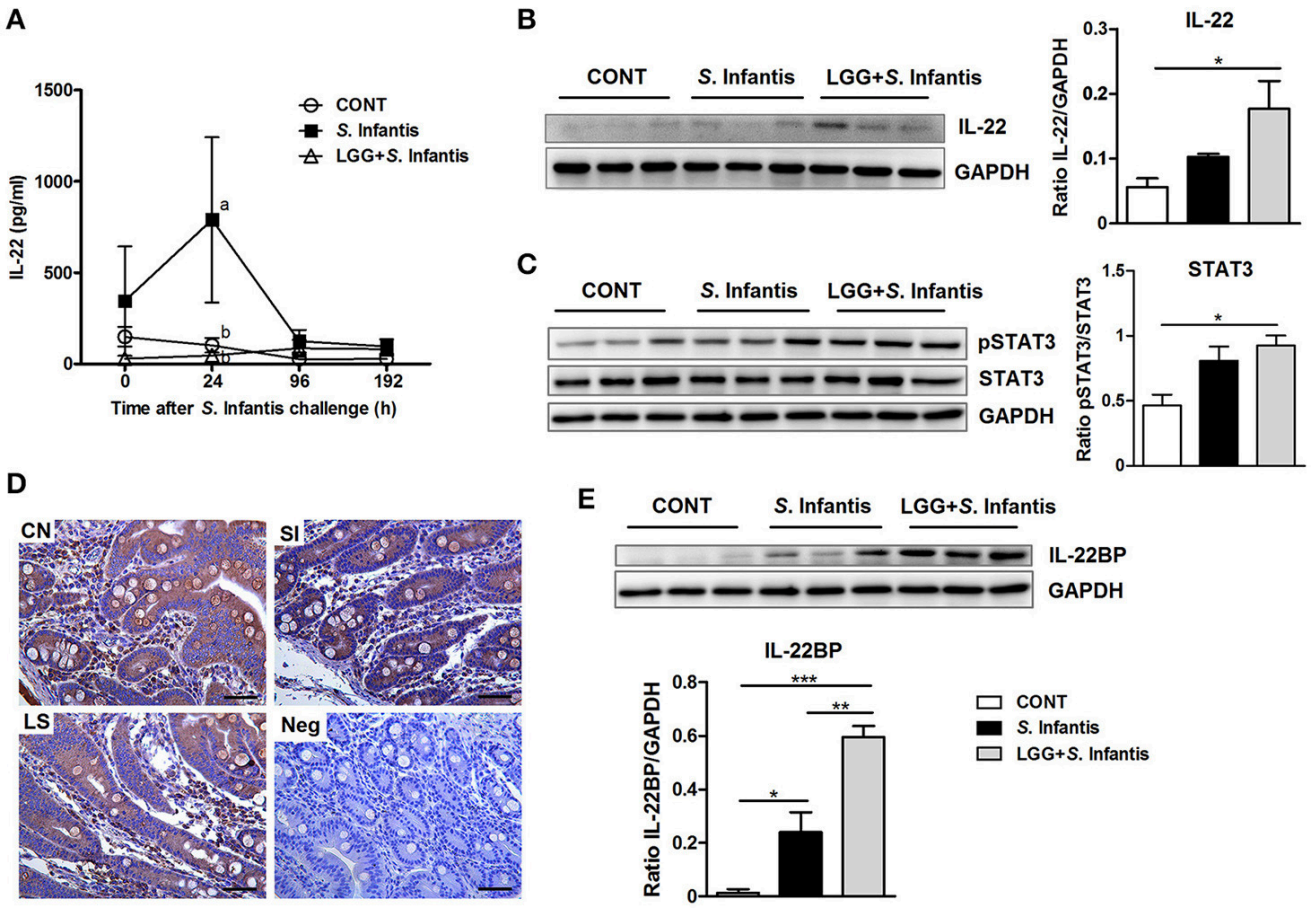
Effect of orally fed LGG on IL-22BP/IL-22/STAT3 expression. **(A)** Serum concentrations of IL-22 were determined by ELISA. Data are presented as the mean ± SEM for each time point (*n* = 6 per group). Mean values at the same time point without a common superscript (^a, b^) differ significantly (*P* < 0.05). Representative Western blot panels of **(B)** IL-22, **(C)** phosphorylation of STAT3 and total STAT3, and **(E)** IL-22BP in ileal tissues collected from pigs 10 days after *S*. Infantis challenge. Each band represents a single pig. Expression of GAPDH was measured as an internal control. Results are presented as the ratio of the intensity of the IL-22 or IL-22BP band to the intensity of the GAPDH band or the ratio of the intensity of the pSTAT3 band to the intensity of the STAT3 band. **(D)** Representative photomicrographs of immunostaining of IL-22BP and negative control using irrelevant mouse serum in jejunal tissues of pigs. IL-22BP-expressing cells in the jejunum were scattered predominantly throughout the lamina propria. Scale bars, 100 μm. Data are expressed in arbitrary units as the mean ± SEM for each tissue (*n* = 6 per group). ^*^P < 0.05; ^**^P < 0.01; ^***^P < 0.001 (Tukey's test).

Levels of IL-22 in the serum of pigs infected only with *S*. Infantis were higher than in CONT pigs at 24 h after *S*. Infantis challenge (*P* = 0.040; Figure 7A). To determine whether LGG induces the activation of IL-22 signaling, we examined the expression of IL-22, pSTAT3, and IL-22BP in the small intestine. The expression of both IL-22 and pSTAT3 was elevated in ileal tissues of pigs pretreated with LGG in comparison with CONT pigs (*P* = 0.038 and *P* = 0.027, respectively; Figures 7B,C). However, this phenomenon was not observed in the jejunum (data not shown). Immunohistochemical analysis revealed that IL-22BP-expressing cells in the small intestine were scattered throughout the lamina propria (Figure 7D). IL-22BP expression increased in the ileum following *S*. Infantis challenge, compared with CONT, including those pretreated with LGG (*P* = 0.042 and *P* < 0.001, respectively; Figure 7E). Unexpectedly, pretreatment with LGG increased IL-22BP levels relative to *S*. Infantis alone (*P* = 0.006; Figure 7E).

### Orally fed LGG suppresses intestinal CCL20 expression in response to *S*. infantis

**Figure 8:**
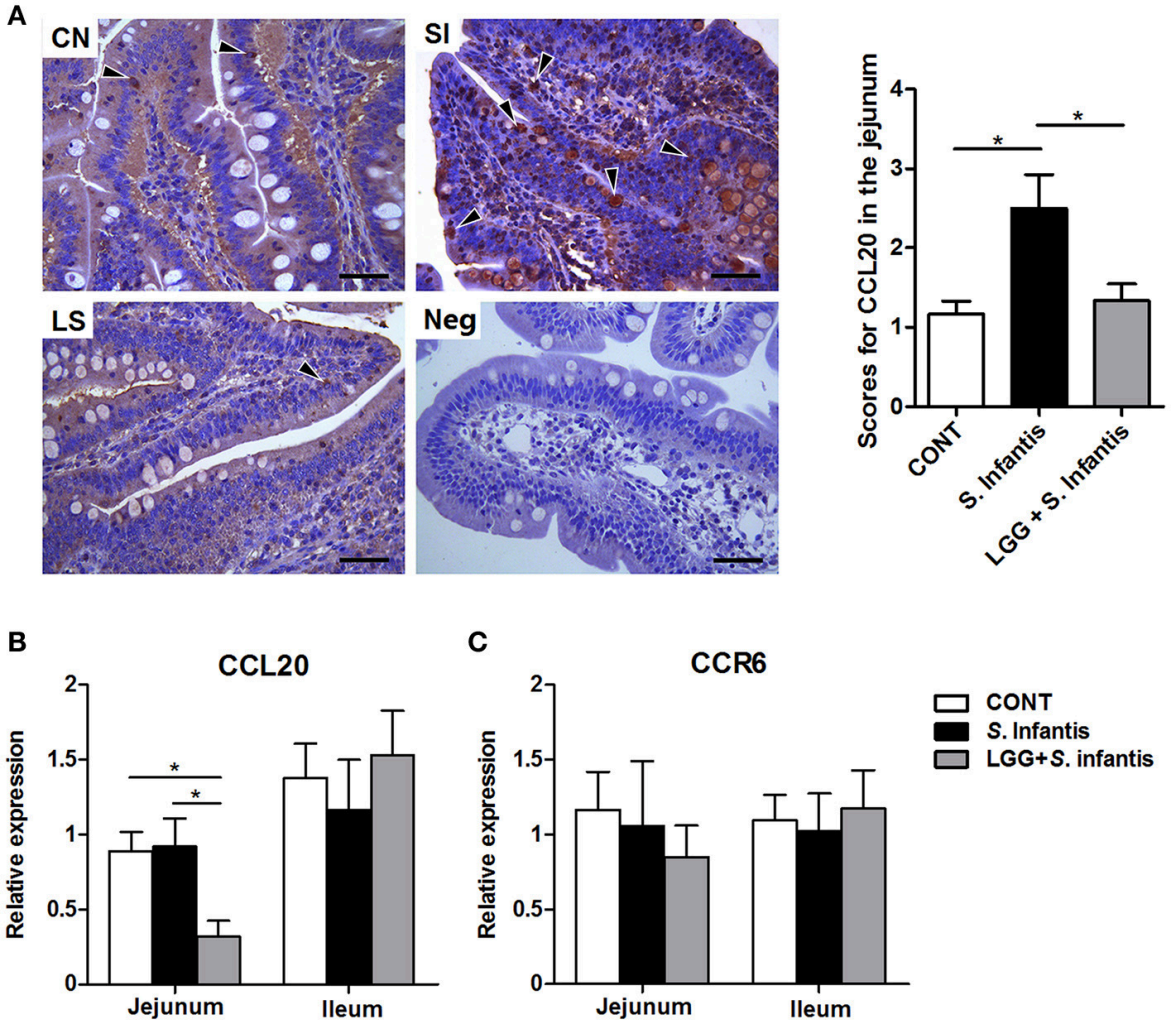
Expression of CCL20/CCR6 in the small intestine. **(A)** Representative photomicrographs of immunostaining of CCL20 and negative control using irrelevant goat serum in jejunal tissues collected from pigs 10 days after *S*. Infantis challenge (left). Arrowheads show that CCL20-positive cells are localized predominantly on the surface epithelium of the villi as well as lamina propria, with sporadic positive staining. Scale bars, 100 μm. The number of CCL20-positive cells was scored (right). The expression of mRNAs for the genes encoding **(B)** CCL20 and **(C)** CCR6 in both jejunal and ileal tissues collected from pigs 10 days after *S*. Infantis challenge was analyzed using quantitative real-time PCR. Data are presented as the mean ± SEM for each tissue (*n* = 7 per group). ^*^P < 0.05 (Tukey's test).

CCL20 was localized primarily at the surface epithelium of the villi but was also present in the lamina propria and goblet cells, as evidenced by sporadic positive staining (Figure 8A). The number of CCL20-positive cells in the jejunum was increased after *S*. Infantis infection compared with the CONT (*P* = 0.015), and this increase was attenuated by LGG pretreatment (*P* = 0.032). Concordantly, the expression of CCL20 mRNA in the jejunum was downregulated in pigs pretreated with LGG compared with CONT pigs and pigs only challenged with *S*. Infantis (*P* = 0.038 and *P* = 0.022, respectively; Figure 8B). However, no changes in CCR6 mRNA expression were observed in the small intestine (Figure 8C).

## Discussion

The emergence of salmonellosis is dependent on an intricate series of host-pathogen interactions affected by the virulence of the *Salmonella* strain and the immunologic status of the host. Our results show that oral inoculation with *S. enterica* serovar Infantis in pigs causes fever, diarrhea, and intestinal inflammation. Of note, pretreatment with the probiotic LGG (1.0 × 10^10^ CFU/day) ameliorated *S*. Infantis-induced inflammation in the jejunum.

Long-term LGG supplementation alters the composition of the enteric microbiota in children, causing a decrease in the abundance of *Escherichia* (Korpela et al., 2016). In the present study, *S*. Infantis infection decreased the number of lactobacilli and *E. coli*, whereas oral administration of LGG increased the abundance of fecal *Bifidobacterium*. LGG pretreatment reduced *Salmonella* colonization of the jejunum, as LGG can compete for adhesion on the surface of intestinal mucosa and excludes *Salmonella* (Alander et al., 1999; Burkholder and Bhunia, 2009). Our study utilizing an *S*. Infantis pig model of infectious enteritis indicates that LGG orally administered to pigs prior to infection diminishes the disease activity, in part by reducing the attachment of pathogens to intestinal mucosa and invasion of the tissues, particularly in the jejunum (Yan et al., 2017).

*S*. Infantis infections are usually limited to the gastrointestinal tract, regardless of the host infected (Volf et al., 2010). As such, colonization by *Salmonella* was only observed in intestinal tissues of piglets and not in the spleen or the liver in our study. The presentation of diarrhea and intestinal inflammation is consistent with our detection in the *S*. Infantis challenge strain of the SPI-1-associated secreted effectors SipA, SopB, SopE, and SopE2, which are responsible for the disruption of tight junction structure and function (Zhang et al., 2003; Boyle et al., 2006). Furthermore, the *S*. Infantis secreted effector SseI might contribute to systemic infection in pigs (McLaughlin et al., 2009).

It has been previously demonstrated that IL-22 levels in the serum of mice are elevated 1 day after *S*. Enteritidis infection (Siegemund et al., 2009). Similarly in the present study, the level of circulating IL-22 was enhanced in pigs after *S*. Infantis infection. IL-22 likely functions in coordinating a general inflammatory response in the serum (Dudakov et al., 2015). Besides lipopolysaccharide, an increase in the IL-22:IL-22BP ratio in inflamed intestine has been postulated as a prominent cause of elevated systemic IL-22 levels (Wolk et al., 2007). By contrast, LGG consumption resulted in a decrease in IL-7 serum concentrations in *S*. Infantis-infected pigs. It has been shown that serum IL-7 levels in pediatric IBD patients with active inflammation were lower vs. those in remission (Kader et al., 2005). Our study revealed that oral administration of LGG attenuates an *S*. Infantis-induced increase in the level of serum IL-22 and excessive Th1 immune responses in peripheral blood, which may contribute to blunting the systemic inflammation caused by *Salmonella*. Collectively, these findings indicate that the fever caused by *S*. Infantis may be associated with lipopolysaccharide and secreted effectors (e.g., SseI) released by the *Salmonella* challenge strain in combination with host systemic proinflammatory cytokines such as IL-22.

IFNγ^hi^ Th1 cells induce *Salmonella* killing more efficiently than IFNγ^lo/−^ Th1 cells (Helmstetter et al., 2015). The production of IFNγ by activated CD4^+^ T cells was increased after stimulation. Our results demonstrate LGG induces the expansion of CD4^+^ T-bet^+^ IFNγ^+^ T cells in peripheral blood, which aids in the eradication of intracellular pathogens (Ravindran et al., 2005). In contrast, *S*. Infantis infection results in a reduction in the proportion of peripheral CD4^+^ T-bet^+^ IFNγ^−^ T cells. T-bet-deficient CD4^+^ T cells (e.g., Th17 cells) can still express substantial amounts of IFNγ (Harms Pritchard et al., 2015; Krausgruber et al., 2016). We found that LGG attenuates the *S*. Infantis-induced expansion of CD4^+^ T-bet^−^ IFNγ^+^ T cells, which could be attributed to the decline in the number of CD4^+^ IFNγ^+^T cells in peripheral blood observed 24 h after infection. T-bet^−^ CD4^+^ T cells promote exacerbated Th17-type inflammatory responses (Krausgruber et al., 2016). However, CD4^+^ T-bet^+^ IFNγ^−^ T cells, which constitute a small proportion of the population, have not been well defined.

In the small intestine, LGG pretreatment attenuated the *S*. Infantis-induced increase in the number of CD4^+^ IFNγ^+^ T cells in PPs and suppressed the expression of IFNγ mRNA in the jejunum. Consistently, probiotic *Lactobacillus* pretreatment suppresses IFNγ mRNA expression in the colon of mice with *Citrobacter rodentium* infection (Rodrigues et al., 2012). LGG prevents IFNγ-induced epithelial barrier dysfunction and inflammation *in vitro* (Donato et al., 2010). Thus these findings indicate that LGG is effective in the prevention of aberrant Th1 responses and excessive IFNγ production that are associated with the progression of inflammation.

It is important to note that levels of T-bet in the intestine were elevated by LGG administration in response to *Salmonella* infection. In line with our result, the expression of T-bet protein in the small intestine was also elevated in immunosuppressed mice treated with *L. plantarum* NCU116 (Xie et al., 2015). T-bet controls the response of mucosal immunity to commensal bacteria, T-bet^−/−^ mice develop more severe colitis (Neurath et al., 2002; Garrett et al., 2007). Coincident with the attenuated histologic features resulting from administration of LGG, the elevated T-bet expression induced by LGG is crucial in resistance to intestinal *S*. Infantis infection. However, the cellular pathways underlying this process remain to be defined.

T-bet has been proposed as a potential suppressor of IL-7Rα expression and IL-7Rα blockade attenuated innate lymphoid cells-mediated IBD (Powell et al., 2012). In *Salmonella*-infected animals, IL-7Rα expression was elevated in the small intestine, whereas the levels of IL-7Rα and IL-7 mRNA declined after oral administration of LGG. IL-7 exacerbates chronic colitis in mice by inducing expansion of mucosal CD4^+^ IL-7R^high^ T cells (Okada et al., 2005). We previously demonstrated that IL-7Rα-expressing cells infiltrated the inflamed ileum in pigs infected with *E. coli*, even with pretreatment of high-dose *Bacillus licheniformis* and *B. subtilis* (BLS-mix) (Yang et al., 2016). In accordance with prior studies, our data demonstrate that probiotic LGG attenuated the upregulation of intestinal IL-7 and IL-7Rα expression induced by *Salmonella* infection, thereby contributing to the amelioration of intestinal inflammation.

Nevertheless, IL-7 is important for the expansion of IL-22-producing cells (Dudakov et al., 2015), and IL-7Rα appears to be required for IL-22 expression in the colon of *C. rodentium*-infected mice (Zhang et al., 2015). We recently found that orally fed probiotic BLS-mix induced the transcription of *IL-22* in the small intestine of pigs infected with *E. coli* (Yang et al., 2016). In the present study, IL-22 production induced by LGG pretreatment further triggered the activation of STAT3, which induced the expression of antimicrobial proteins to defend the mucosal surfaces from invading pathogens (Backert et al., 2014). Activation of STAT3 ameliorates intestinal inflammation and prevents systemic dissemination of bacteria in mice with *C.rodentium* infection (Wittkopf et al., 2015). It has been found that lactobacilli induce IL-22 production *ex vivo* in mouse stomach via the metabolite indole-3-aldehyde (Zelante et al., 2013). Therefore, the probiotic active component derived from LGG should be examined in more detail in the future.

Interestingly, LGG also promoted intestinal IL-22BP production following *S*. Infantis challenge. Consistent with mouse infectious models, the expression of IL-22BP mRNA was upregulated in the liver after *Mycobacterium tuberculosis* infection (Wilson et al., 2010). The overproduction of IL-22BP in the inflamed mucosa may block IL-22 protective actions (Martin et al., 2016). But meanwhile the regulation of IL-22BP expression is crucial for controlling the activity of IL-22-mediated intestinal tissue damage, especially during later stages of *Salmonella* infection. IL-22BP was discovered helpful to counteract IL-22-mediated proinflammatory responses during acute polymicrobial peritonitis in mice (Weber et al., 2007). In agreement with this, orally administered LGG attenuated inflammatory enteritis induced by *S*. Infantis infection. From our data, we hypothesize that high IL-22BP expression induced by LGG plays a role in limiting IL-22-elicited proinflammatory responses during *S*. Infantis infection.

Increased IL-22 levels might inhibit the expression of CCL20 in gastric epithelial cells and thus protect the gastric mucosa from inflammation-mediated damage (Chen et al., 2014). As expected (Sibartie et al., 2009; Toki et al., 2009), we found that LGG suppressed the production of CCL20 in the jejunum induced by oral infection with *S*. Infantis, although no changes in CCR6 mRNA expression in the small intestine were observed. It has been reported the expansion of goblet cells led to the downregulation of CCL20 in follicle-associated epithelium (Obata et al., 2014). CCL20 expression can be promoted by producing the proinflammatory cytokines TNF-α and IL-1β during *Salmonella* infection (Sugita et al., 2002). Taken together, LGG decreases the expression of inflammatory CCL20 in the jejunum in response to *S*. Infantis infection, thereby contributing to the amelioration of *Salmonella*-induced inflammation.

**Figure 9:**
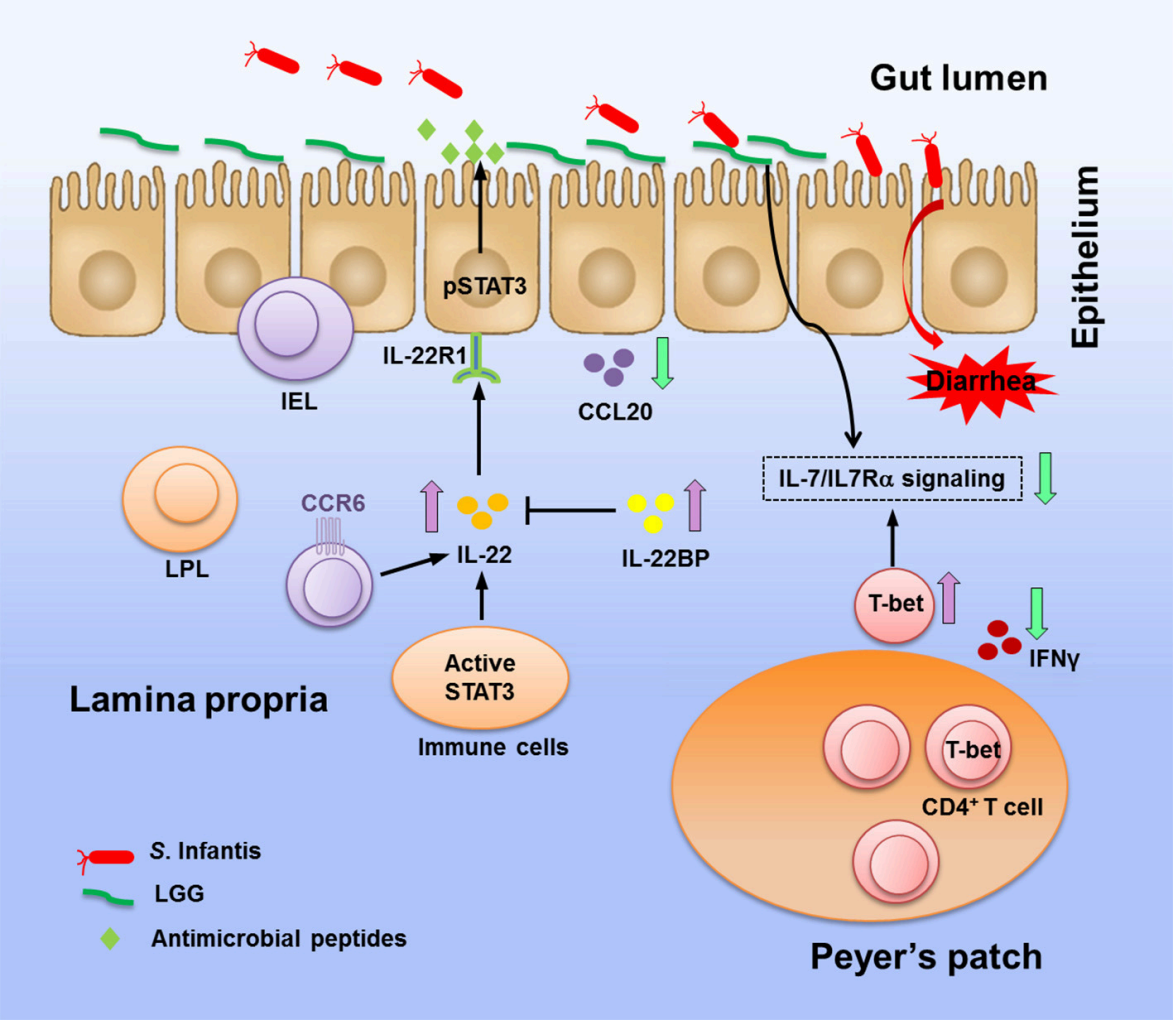
Coordinating reactions in the intestine elicited by LGG in response to *S*. Infantis infection. Oral infection with *Salmonella* Infantis causes diarrhea in newly weaned piglets. LGG pretreatment prevents colonization of the jejunum by *Salmonella*, thereby blunting pathogen invasion. In response to *S*. Infantis infection, LGG enhances the expression of IL-22 and STAT3 activity, thus inducing the production of antimicrobial proteins to protect mucosal surfaces from invading pathogens. Elevation of IL-22BP expression is significant in prevention of the IL-22-associated proinflammatory damage to tissues. LGG suppresses the expression of the inflammatory chemokine CCL20, which attracts CCR6^+^ cells to tissues. LGG downregulates the expression of IL-7 and IL-7Rα but elevates the expression of T-bet, which has been proposed as a suppressor of IL-7Rα (Powell et al., 2012). LGG is potential in preventing aberrant CD4^+^ IFNγ^+^ T cell responses and excessive IFNγ production in the PPs caused by intracellular bacteria. The above activity of LGG against *Salmonella* and its regulation of immune responses during *S*. Infantis infection contribute to the amelioration of intestinal inflammation.

In conclusion, our study in a pig model indicates that oral administration of probiotic LGG confers a degree of protection against *S*. Infantis infection. Our results suggest that LGG excludes pathogens and promotes mucosal immunity to *Salmonella* infection through expansion of CD4^+^ T-bet^+^ IFNγ^+^ T cells, elevation of T-bet levels, and activation of the IL-22BP–IL-22–STAT3 pathway. Additionally, LGG pretreatment suppresses CCL20 expression and attenuates the upregulation of IL-7Rα expression in response to *S*. Infantis infection, contributing to the amelioration of intestinal inflammation and maintenance of mucosal homeostasis (Figure 9). These observations provide potentially relevant insights for clinical trials aimed at screening effective targets for salmonellosis therapy.

## Author contributions

GY participated in the study design, performed the experiments, analyzed the data, and wrote the manuscript; JY performed the bacterial cultures and bacterial sequence analyses; JS performed the flow cytometry analyses; LJ performed bacterial isolation and preparation; XL performed the immunofluorescence assay; YZ conceived and designed the study.

### Conflict of interest statement

The authors declare that the research was conducted in the absence of any commercial or financial relationships that could be construed as a potential conflict of interest.

## Abbreviations

*S*. Infantis: *Salmonella enterica* serovar Infantis
LGG: *Lactobacillus rhamnosus* GG
IL: Interleukin
IL-22BP: IL-22-binding protein
IEL: intraepithelial lymphocyte
LPL: lamina propria lymphocyte
PPL: Peyer's patch lymphocyte
IL-7Rα: IL-7 receptor α-chain
CCL: CC-chemokine ligand
CCR: CC-chemokine receptor.
