# Supplementary material for: Oral Administration of Lactobacillus rhamnosus GG Ameliorates Salmonella Infantis-Induced Inflammation in a Pig Model via Activation of the IL-22BP/IL-22/STAT3 Pathway
Source: Front Cell Infect Microbiol. 2017 Jul 18;7:323. doi: 10.3389/fcimb.2017.00323 (PMC5514694; doi:10.3389/fcimb.2017.00323)
Supplement: Supplementary file 1 [file Table1.DOC]

**Table S1 Primers used for the detection of virulence genes by real-time PCR**

| **Gene**  **product** | **Primer** | | **Product**  **size (bp)** | **Accession number** |
| --- | --- | --- | --- | --- |
|  | **Direction*****^a^*** | **Sequence (5'→3')** |  |  |
| SipA | F | GTCATTCGCGTGTGGATTCG | 143 | CP016410.1 |
|  | R | TTCGGATGAAGCGTTGGTCA |  |  |
| SipB | F | CGTTGTGGCCGCTGTTTTTA | 222 | CP016410.1 |
|  | R | GCCGTTTTCTTATCGACGCC |  |  |
| SopA | F | CTCTCCACTGACACGCTGTT | 168 | CP016410.1 |
|  | R | GGCCTGCTGATTAAATGCGG |  |  |
| SopB | F | CTTATGAGGGAAAGGGCG | 86 | AE006468 |
|  | R | ATGCACACTCACCGTGG |  |  |
| SopD | F | TTCGAAGATGACCTGGCACC | 155 | CP016410.1 |
|  | R | GTGAGTCCTGCCATTCGACA |  |  |
| SopE | F | GATCCCACGAATCTCCACCC | 182 | AF043239.1 |
|  | R | ATGGGCTGCAGGAATTCGATA |  |  |
| SopE2 | F | ACGGTAACGACACCTGCATT | 106 | AF217274.1 |
|  | R | TCCGATCTCGAGACATAAACCA |  |  |
| FljA | F | TTCATTAGGTCCCCTCCGGT | 176 | AE006468.2 |
|  | R | CAGGCGTAAATGCGTGTCAG |  |  |
| FljB | F | GCTCCTGTCGCTTCATCGTA | 277 | CP016410.1 |
|  | R | GTACAGTAACCCTTGCGGCT |  |  |
| SseI | F | ATTTATCGTATTGCCTGGTC | 200 | CP016410.1 |
|  | R | TCCTCCCATCCGTCATAC |  |  |

*^a^* F = forward; R = reverse.
