# Supplementary material for: Oral Administration of Lactobacillus rhamnosus GG Ameliorates Salmonella Infantis-Induced Inflammation in a Pig Model via Activation of the IL-22BP/IL-22/STAT3 Pathway
Source: Front Cell Infect Microbiol. 2017 Jul 18;7:323. doi: 10.3389/fcimb.2017.00323 (PMC5514694; doi:10.3389/fcimb.2017.00323)
Supplement: Supplementary file 2 [file Table2.DOC]

**Table S2 Sequences of oligonucleotide primers used for quantitative real-time PCR, length of the respective PCR product, and gene accession numbers.**

| **Gene**  **product*^a^*** | **Primer** | | **Product**  **size (bp)** | **Accession number** |
| --- | --- | --- | --- | --- |
|  | **Direction*****^b^*** | **Sequence (5'→3')** |  |  |
| HPRT | F | GTGATAGATCCATTCCTATGACTGTAGA | 104 | U69731 |
|  | R | TGAGAGATCATCTCCACCAATTACTT |  |  |
| GAPDH | F | CCAGAACATCATCCCTGCTT | 229 | NM_001206359.1 |
|  | R | GTCCTCAGTGTAGCCCAGGA |  |  |
| β-actin | F | CTCTTCCAGCCCTCCTTCCT | 103 | XM_003357928.2 |
|  | R | GCGTAGAGGTCCTCCTGATGT |  |  |
| IFNγ | F | TAAATGGTAGCTCTGGGAAACTGAA | 86 | NM_213948 |
|  | R | GATGGCTTTGCGCTGGA |  |  |
| CCL20 | F | GCTCCTGGCTGCTTTGATGTC | 143 | XM_005672261.2 |
|  | R | CATTGGCGAGCTGCTGTGTG |  |  |
| CCR6 | F | CGGAACATCACCGAGGTCC | 80 | DQ991099.1 |
|  | R | CGGAACTTCTGCCCGATGAA |  |  |
| IL-7 | F | AGCAATTGCCTGAATAACGAACC | 223 | NM_214135.2 |
|  | R | CACCCAGGGAAGGTGGTTTT |  |  |

*^a^* HPRT, hypoxanthine phosphoribosyl-transferase; GAPDH, glyceraldehyde-3-phosphate dehydrogenase; CCL, CC-chemokine ligand; CCR, CC-chemokine receptor ; IL, interleukin.

*^b^* F, forward; R, reverse.
