# Supplementary material for: Oral Administration of Lactobacillus rhamnosus GG Ameliorates Salmonella Infantis-Induced Inflammation in a Pig Model via Activation of the IL-22BP/IL-22/STAT3 Pathway
Source: Front Cell Infect Microbiol. 2017 Jul 18;7:323. doi: 10.3389/fcimb.2017.00323 (PMC5514694; doi:10.3389/fcimb.2017.00323)
Supplement: Supplementary file 3 [file Table3.DOC]

**Table S3 Effects of** **oral administration of** ***Lactobacillus rhamnosus* GG on the incidence** **of diarrhea in newly weaned pigs before and after** ***S*. Infantis challenge.**

| **Group*^a^*/item** | **Pigs** |  |  |  | |  | **Pig days** | |  |  |  |  |
| --- | --- | --- | --- | --- | --- | --- | --- | --- | --- | --- | --- | --- |
|  | **At risk** | **With diarrhea** | | | **At risk** | | **With diarrhea** | |  | **Significance of difference** | | |
|  | **(n)** | **(n)** | **(%)** |  | | **(n)** | **(n)** | **(%)** | ***S*. infantis** **LGG+*S*.infantis** | | | |
| Before infection |  |  |  |  | |  |  |  |  |  |  |  |
| CONT | 7 | 4 | 57.14 |  | | 49 | 9 | 18.36 |  |  | ** |  |
| *S*. Infantis | 7 | 1 | 14.28 |  | | 49 | 6 | 12.24 |  |  |  |  |
| LGG+*S*. Infantis | 7 | 2 | 28.57 |  | | 49 | 5 | 10.20 |  |  |  |  |
|  |  |  |  |  | |  |  |  |  |  |  |  |
| Post-infection |  |  |  |  | |  |  |  |  |  |  |  |
| CONT | 7 | 0 | 0 |  | | 70 | 0 | 0 |  | *** | ** |  |
| *S*. Infantis | 7 | 4 | 57.14 |  | | 70 | 10 | 14.28 |  |  |  |  |
| LGG+*S*. Infantis | 7 | 3 | 42.85 |  | | 70 | 7 | 10.00 |  |  |  |  |

*^a^* Piglets received sterile physiologic saline orally (CONT), received sterile physiologic saline orally followed by *S*. Infantis (5.0 × 10^10^ CFU/ml, 10 ml, p.o.) challenge (*S*. Infantis), or were pretreated with *Lactobacillus rhamnosus* GG (LGG, 1.0 × 10^9^ CFU/ml, 10 ml once daily, p.o.) for 1 week followed by *S*. Infantis challenge (LGG + *S*. Infantis).

n = 7 pigs per group; Pearson’s chi-square test.
